# Organoid modeling of lung-resident immune responses to SARS-CoV-2 infection

**DOI:** 10.21203/rs.3.rs-2870695/v1

**Published:** 2023-05-05

**Authors:** Shannon S. Choi, Vincent van Unen, Huimin Zhang, Arjun Rustagi, Samira A. Alwahabi, António J.M. Santos, Joshua E. Chan, Brandon Lam, Daniel Solis, Jordan Mah, Katharina Röltgen, Winston Trope, Alexander Guh-Siesel, Zhongqi Lin, Aimee Beck, Caitlin Edwards, Vamsee Mallajosyula, Brock A. Martin, James C. Y. Dunn, Joseph Shrager, Ralph A. Baric, Benjamin Pinsky, Scott D. Boyd, Catherine A. Blish, Mark M. Davis, Calvin J. Kuo

**Affiliations:** 1 Department of Medicine, Divisions of Hematology, Stanford University School of Medicine, Stanford, CA 94305, USA; 2 Departments of Microbiology and Immunology, Stanford University School of Medicine, Stanford, CA 94305, USA; 3 Stanford Institute of Immunity, Transplantation and Infection, Stanford University School of Medicine, Stanford, CA 94305, USA; 4 Department of Infectious Disease and Geographic Medicine, Stanford University School of Medicine, Stanford, CA 94305, USA; 5 Department of Pathology, Stanford University School of Medicine, Stanford, CA 94305, USA; 6 Department of Cardiothoracic Surgery, Stanford University School of Medicine, Stanford, CA 94305, USA; 7 Department of Microbiology and Immunology, University of North Carolina Chapel Hill, Chapel Hill, NC 27599, USA; 8 Department of Pediatric Surgery, Stanford University School of Medicine, Stanford, CA 94305, USA; 9 Chan Zuckerberg Biohub, San Francisco, CA 94158, USA; 10 Howard Hughes Medical Institute, Stanford University School of Medicine, Stanford, CA 94305, USA

## Abstract

Tissue-resident immunity underlies essential host defenses against pathogens, but analysis in humans has lacked *in vitro* model systems where epithelial infection and accompanying resident immune cell responses can be observed *en bloc*. Indeed, human primary epithelial organoid cultures typically omit immune cells, and human tissue resident-memory lymphocytes are conventionally assayed without an epithelial infection component, for instance from peripheral blood, or after extraction from organs. Further, the study of resident immunity in animals can be complicated by interchange between tissue and peripheral immune compartments. To study human tissue-resident infectious immune responses in isolation from secondary lymphoid organs, we generated adult human lung three-dimensional air-liquid interface (ALI) lung organoids from intact tissue fragments that co-preserve epithelial and stromal architecture alongside endogenous lung-resident immune subsets. These included T, B, NK and myeloid cells, with CD69^+^CD103^+^ tissue-resident and CCR7^−^ and/or CD45RA^−^ T_RM_ and conservation of T cell receptor repertoires, all corresponding to matched fresh tissue. SARS-CoV-2 vigorously infected organoid lung epithelium, alongside secondary induction of innate cytokine production that was inhibited by antiviral agents. Notably, SARS-CoV-2-infected organoids manifested adaptive virus-specific T cell activation that was specific for seropositive and/or previously infected donor individuals. This holistic non-reconstitutive organoid system demonstrates the sufficiency of lung to autonomously mount adaptive T cell memory responses without a peripheral lymphoid component, and represents an enabling method for the study of human tissue-resident immunity.

## INTRODUCTION

Tissue-resident immunity represents an essential line of defense against infection. In the lung, innate and adaptive immunity coordinately respond to pathogens, with resident memory lymphocytes mediating rapid recall responses^1,2^. However, the study of human lung-resident memory responses has been complicated by the relative inaccessibility of pulmonary tissue for experimental purposes, and lack of *in vitro* epithelialimmune experimental systems, while mouse systems lack human context and possess obligate interchange between tissue and peripheral lymphoid compartments^1–3^. It is further unclear if organs such as lung are themselves sufficient to implement memory immune responses in the absence of interaction with secondary lymphoid organs^4^. In the current COVID-19 pandemic, SARS-CoV-2 prompts a coordinated innate and adaptive immune response involving both peripheral and tissue compartments, characterized by production of cytokines and antibodies, as well as T cell activation^5–7^. Continued pandemic waves have highlighted both the persistence and deficiencies of SARS-CoV-2 adaptive immune responses, typically measured in peripheral blood^8–10^. Yet, the ability of lung-resident lymphocytes to mediate mucosal immunity against SARS-CoV-2, to embody crucial memory defenses against repeated infection, and to elicit pathologic inflammation as in acute respiratory distress syndrome and cytokine storm^11^, all emphasize the need for relevant experimental systems.

Organoids have recently emerged as *ex vivo* models for infectious disease research^12^. We and others previously modeled SARS-CoV-2 infection in lung organoids comprised exclusively of epithelium, including alveolar type 2 (AT2), ciliated and club cells^13–19^, but such systems typically lack tissue-resident immune populations, hampering investigations of inflammation during pathogenesis. Conversely, mononuclear fractions from peripheral blood or lung parenchyma allow study of SARS-CoV-2 memory responses, but omit viral infection of epithelium^3,20–23^, while short-lived lung explants exhibit limited viability^24^. Alternatively, rodent COVID-19 models are limited by interspecies host-pathogen incompatibility^25,26^ and immortalized cell lines do not accurately recapitulate the cellular diversity and physiology of primary tissue^27,28^. A robust human lung primary culture system that allows pathogen infection while holistically preserving epithelium, mesenchyme and tissue-resident immune cells has been notably lacking.

Here, we extended an air-liquid-interface (ALI) method previously used to model cancer progression and the tumor microenvironment^29–31^ to develop and characterize novel 3D human distal lung organoids containing not only multiple epithelial cell types but also an extensive complement of mesenchymal stroma and functional tissue-resident immune cells. This contrasted with peripheral blood lymphocyte reconstitution approaches^32^ and commonly employed 2D monolayer ALI cultures exclusively comprised of lung epithelium^15,33,34^. The resultant 3D ALI organoids demonstrated long-term co-preservation and proliferation of native lung epithelial and immune components and allowed study of pulmonary immunity to SARS-CoV-2 in the absence of peripheral lymphoid tissue. The application of this holistic organoid system to study SARS-CoV-2 further demonstrated the existence of a functional, lung-intrinsic adaptive immune response to pathologic infection.

## RESULTS

### Human lung 3D ALI organoids grow robustly in culture and preserve distal lung architecture

**Figure 1.**
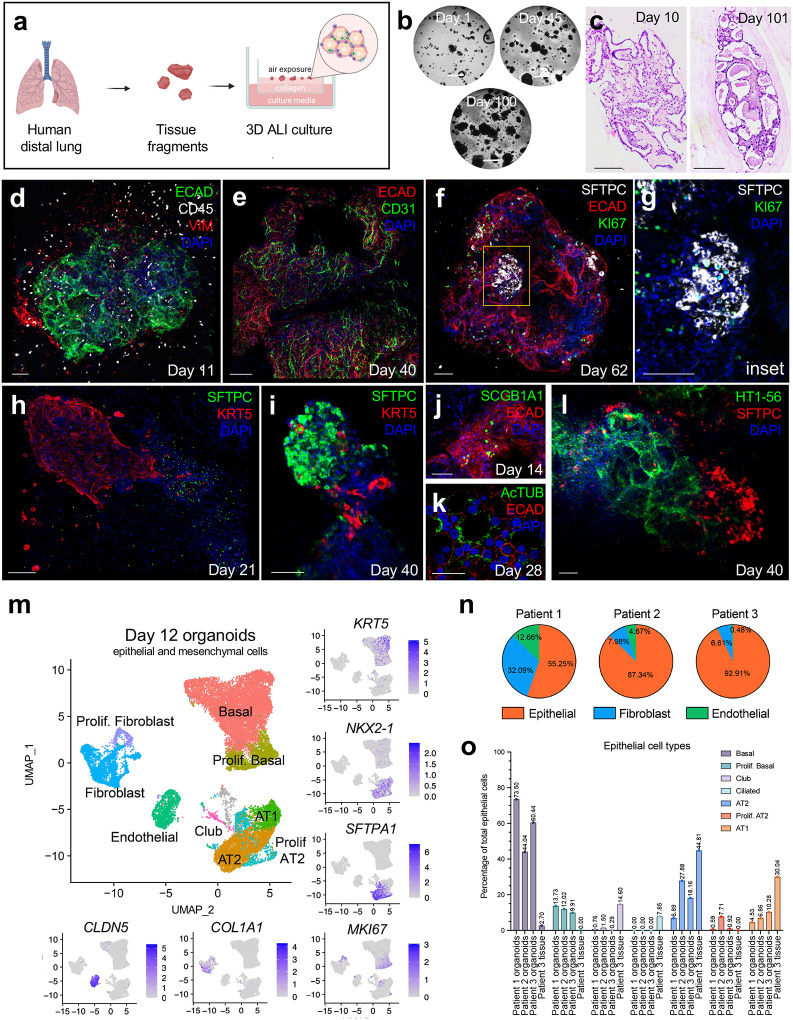
Characterization of epithelial and mesenchymal cells in lung air-liquid-interface organoids. **a,** Schematic of lung air-liquid-interface organoid generation. **b,** Serial brightfield images of lung ALI organoids, 1–100 days. Scale bar, 2 mm. **c,** Hematoxylin and eosin (H&E) staining of lung ALI organoids, day 10 and day 101. Scale bar, 500 μm. **d,** Immunofluorescence (IF) staining of day 11 lung organoid, ECAD^+^ epithelial (green), CD45^+^ immune (white), and Vimentin^+^ (VIM) mesenchymal cells (red), DAPI (blue). Scale bar, 100 μm. **e,** IF staining of day 40 lung organoid, ECAD^+^ epithelial (red) and CD31^+^ endothelial cells (green), DAPI (blue). Scale bar, 100 μm. **f,** IF staining of day 62 lung organoid, SFTPC^+^ AT2 cells (white), ECAD (red), KI67 (green), DAPI (blue). Scale bar, 100 μm. **g,** Inset of yellow region from (f) showing proliferative AT2 cells, SFTPC (white), KI67 (green), DAPI (blue). Scale bar, 300 μm. **h,** IF staining of day 21 organoid depicting SFTPC^+^ AT2 cells (green) and KRT5^+^ basal cells (red), DAPI (blue). Scale bar, 100 μm. **i,** IF staining of day 40 organoid showing SFTPC^+^ AT2 cells (green) and KRT5^+^ basal cells (red), DAPI (blue). Scale bar, 100 μm. **j,** IF staining of day 14 organoid showing SGB1A1^+^ club cells (green) ECAD (red), DAPI (blue). Scale bar, 100 μm. **k,** IF staining of day 28 organoid with AcTub^+^ ciliated cells (green) ECAD (red), DAPI (blue). Scale bar, 100 μm. **l,** IF staining of day 40 organoid with HT1–56^+^ AT1 cells (green) SFTPC^+^ AT2 cells (red), DAPI (blue). Scale bar, 100 μm. **m,** scRNA-seq UMAP plot and feature plots integrating CD45^−^ cells from organoids of three individuals. **n,** Pie charts showing proportions of epithelial, fibroblast, and endothelial cells from scRNA-seq data from (m). **o,** Proportions of epithelial cell types out of total epithelial cells from (m) with 3 different patients (two solely with organoids, one matched organoid-tissue pair).

The cellular composition of the human lung encompasses diverse epithelial cell types, mesenchymal cells such as fibroblasts and endothelial cells, and a dynamic tissue-resident immune landscape. Prior epithelial-only lung organoids have been grown from single cells of enzymatically dissociated lung or cell-type specific subfractions and submerged in extracellular matrix beneath culture media^13,17,18^. Yet, such exclusively epithelial organoids do not model the adaptive immune responses that are critical in pathogen restriction and modulation of inflammation. We thus adapted an organotypic 3D culture system that propagates intact tissue fragments within a non-submerged collagen gel-containing transwell under an air-liquid interface (ALI), allowing direct air exposure from above, where endogenous tissue-infiltrating immune cells are preserved^29,30,35^ (Fig. 1a). This 3D ALI approach contrasts with conventional 2D ALI monolayer cultures of lung ciliated epithelium^33,34^. We accordingly used 3D ALI to propagate tissue fragments from non-cartilaginous distal lung regions containing alveoli and terminal bronchioles in culture media containing EGF, Noggin, and the GSK-3 inhibitor CHIR-99021. Compared to submerged models, the ALI allowed increased oxygenation^36^ while lack of enzymatic dissociation preserved intact tissue structure and architecture. From normal distal lung tissue of over 80 patients undergoing surgical lobectomies and wedge resections, lung ALI organoids expanded in culture for up to 180 days (longest time attempted) with >80% success rate (Fig. 1b, **Extended Data Fig. 1a)**. Typically, organoids preserved the cuboidal epithelium lining of alveolus-like spaces and bronchiolar elements interspersed by extracellular matrix (Fig. 1c **left, Extended Data Fig. 1b)**. At extended time points, epithelial architecture persisted (Fig. 1c **right)**, albeit with increasing extracellular matrix deposition.

### Distal lung ALI organoids maintain diverse epithelial and mesenchymal composition

We next used immunofluorescence to further characterize the cellular composition of these human lung ALI organoids. E-cadherin^+^ (ECAD) epithelial cells were closely associated with vimentin^+^ (VIM) mesenchyme and were interspersed with CD45^+^ hematopoietic cells (Fig. 1d). EdU incorporation into day 12 organoids revealed proliferative indices for epithelial, immune, and mesenchymal compartments of 10%, 6% and 10%, respectively **(Extended Data Fig. 1c)**. CD31^+^ endothelial cells formed networks within organoids (Fig. 1e). SFTPC^+^ AT2 cells grew in distinct KI67^+^ clusters for over 60 days (Fig. 1f, g). Like AT2 cells, KRT5^+^ basal cells also grew in defined areas as bronchiole-like structures extending into AT2 rich alveolar domains (Fig. 1h, i). Although spatially separated, AT2 cells and basal cells co-existed within the same large organoids, contrasting with mutually exclusive epithelial-only basal or alveolar organoids^13^. Additionally, ALI organoids contained rare SCGB1A1^+^ club cells (Fig. 1j). Compared to club cells, acetylated-tubulin^+^ ciliated cells only appeared after approximately three weeks (Fig. 1k), recalling the delayed time kinetics of ciliagenesis in epithelial-only distal lung organoids^13^. Lastly, lung ALI organoids also contained alveoli-like cystic structures lined with HT1–56^+^ AT1 cells. SFTPC^+^ AT2 cells could be observed with AT1 cells but were typically present in adjacent regions (Fig. 1l). All epithelial cell types persisted over extended time points (60–101 days), while maintaining the adjacency of AT2 and basal cell domains **(Extended Data Fig. 1d-g)**.

Single-cell RNA sequencing was used to further characterize organoid epithelial and mesenchymal populations in FACS-sorted day 12 organoids from three patients, in a CD45-negative fraction that excluded hematopoietic elements. Multi-patient data were integrated for phenotyping and UMAP visualization (Fig. 1m). The proportion of *EPCAM*^+^ epithelial cells compared to *COL1A1*^+^ fibroblasts and *CLDN5*^+^ endothelial cells varied in the three individuals with epithelium consistently comprising the majority (Fig. 1n). Basal cells were phenotyped as a *KRT5*^+^ population that contained an *MKI67*^+^ proliferative subfraction, while *SCGB1A1*^+^ club cells were also observed **(Extended Data Fig. 1h).** AT2 cells co-expressed the lung epithelial cell marker *NKX2-*1 with the AT2 markers *SFTPA1, SFTPC and LAMP3*^18,37^, while AT1 cells co-expressed *NKX2–1*, the AT1 marker *AQP5* and lacked *SFTPC/LAMP3*^37,38^ **(Extended Data Fig. 1h).** Ciliated cells, which appeared after 3 weeks, were absent at the 12-day time point. In contrast, patient-matched fresh tissue contained ciliated cells but was essentially devoid of *MKI67*^+^ proliferative fractions (Fig 1o, **Extended Data Fig. 2)**.

### Human lung ALI organoids retain diverse immune cell populations

**Figure 2.**
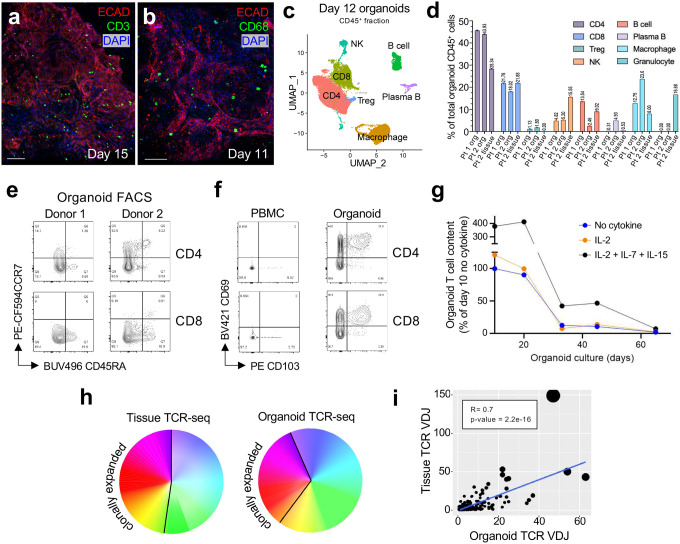
Lung ALI organoids preserve diverse functional immune populations. **a,** IF imaging of day 15 ALI organoids containing T cells. CD3 (green), ECAD (red), DAPI (blue). Scale bar, 100 μm. **b,** IF imaging of day 11 organoids containing macrophages. CD68 (green), ECAD (red), DAPI (blue). Scale bar, 100 μm. **c,** scRNA-seq UMAP plot integrating CD45^+^ cells from lung organoids of two individuals. **d,** Proportions of immune cell types as a fraction of total immune cells from scRNA-seq data of CD45^+^ cells from two different patients (one organoid, one matched organoid-tissue pair). **e,** Organoid T cells exhibit a predominant memory phenotype. Flow cytometry plots of day 12 ALI organoids for CCR7 and CD45RA, pre-gated on live, single, CD45^+^CD3^+^ T cells. **f,** Flow cytometry plots of PBMC versus day 12 ALI organoids for residency markers CD69 and CD103/ITGAE, pre-gated on live, single, CD45^+^CD3^+^ T cells. **g,** Organoid T cell content over 10, 20, 33, and 44 days in culture with values with addition of cytokines as a percentage of the day 10 no cytokine condition. **h,** Pie chart showing individual TCR clones from scRNA-seq/TCR-seq of a matched fresh distal lung/organoid pair. Each color represents an individual TCR clonotype and clonally expanded TCR sequences are represented by large domains of a single color. **i,** Line graph of clonally expanded TCRs from (h). Each dot represents a unique TCR clonotype determined by single cell TCR-seq and the dot size represents relative TCR count frequencies within organoid or cognate fresh tissue. Pearson’s correlation coefficient R= 0.7, p-value = 2.2 × 10^−16^.

We next characterized the immune cell content of the lung ALI organoids. Anti-CD3 and -CD68 immunofluorescence revealed T cells and macrophages embedded throughout the epithelium (Fig 2a, b). Single cell RNA sequencing of live, CD45^+^ FACS-sorted single cells from dissociated organoids, representing a pan-hematopoietic fraction from three separate patients at day 12, identified *CD4*^+^ T, *CD8A*^+^ T, *MS4A1*^+^ B cell, and *CD68*^+^ macrophage populations (Fig. 2c–d). These cell populations were verified by flow cytometry on cell surface markers, where NK cells (*NKG7*^+^) cells, Treg (*CD3E*^+^ *CD4*^+^*, FOXP3*^+^) and plasma cells (*SDC1*^+^) were also observed **(Extended Data Fig. 3a-c)**. Organoid T cells were consistent with resident memory cells (T_RM_) as they overwhelmingly displayed a memory phenotype with absent CCR7 and/or CD45RA, a paucity of naïve CCR7^+^CD45RA^+^ subsets (Fig. 2e) and expressed tissue residency markers CD69 and CD103 upon scRNA-seq and FACS (Fig. 2f, **Extended Data Fig. 3d)**. The CD45^+^ fraction from fresh peripheral lung tissue and corresponding ALI organoids revealed preservation of all major immune cell types except short-lived granulocytes (Fig. 2d, **Extended Data Fig. 3e-f)**. Immune subsets were consistently represented in organoids from all subjects, although individual variation in the specific immune composition was recognized.

Initially, organoid immune infiltrates consisted mostly of lymphocytes, with a predominance of CD3^+^CD4^+^ T cells, followed by CD3^+^CD8^+^ T cells and rarer CD3^−^CD19^+^ B cells. Without exogenous cytokine support, lung ALI organoid lymphocytes decreased substantially over time, with virtually none remaining at day 33 and beyond, consistent with ALI tumor organoids^35^. In contrast, recombinant IL-2, IL7, and IL-15 supplementation enabled an initial 4-fold T cell expansion with preservation at 46% of the day 7 level by 44 days, dissipating by 65 days (Fig. 2g). SSC^hi^, CD68^+^, CD11C^−^ macrophages comprised a minority of organoid CD45^+^ cells that persisted over time without cytokine supplementation **(Extended Data Fig. 3g)**. Organoid culture accurately recapitulated the T cell receptor (TCR) repertoire of matched fresh lung tissue at day 12, as determined by singe cell 5’ VDJ TCR sequencing (Pearson’s correlation coefficient R= 0.7, p = 2.2 × 10^−16^) (Fig. 2h–i, **Extended Data Fig. 4a).** Notably, TCR clonotypes conserved between fresh tissue and organoid matched known sequences recognizing viral pathogens, including SARSCoV-2, influenza, CMV and EBV **(Extended Data Fig. 4b, c)**.

Within the intact lung organ microenvironment, MHC class II is broadly expressed in pulmonary epithelial and immune cell types. Additionally, MHC-II-expressing SPC^+^ AT2 cells can internalize and present peptide and full-length protein antigens, and mouse lung epithelial deletion of *H2Ab1* compromises lung-resident T_RM_ function and pathogen response^39,40^. Upon FACS and scRNA-seq analysis, MHC-II was expressed by diverse ALI lung organoid populations, including epithelium (AT2 and club), endothelium, and B lymphocytes, mirroring MHC-II expression in cognate fresh tissue except for additional MHC-II expression in macrophages **(Extended Data Fig. 5a, b)**. Further, lung ALI organoid EPCAM^+^ epithelial cells avidly internalized and proteolytically degraded DQ-Ovalbumin (DQ-OVA)^48^ to release the quenched BODIPY fluorophore dye intracellularly, formally demonstrating antigen processing function intrinsic to ALI lung organoids **(Extended Data Fig. 5c)**. *TAP1*, involved in MHC-I antigen presentation, was also broadly expressed in organoid epithelial and immune compartments **(Extended Data Fig. 5b).**

### Suspension culture allows epithelial/stromal reorientation and apical ACE2 access

The SARS-CoV-2 entry receptor ACE2 is expressed on the apical surface of lung epithelium^41^, which is typically oriented towards the central lumen in epithelial-only organoids and inconveniently precludes access by virus added to the culture medium^13^. In simple epithelium-only lung organoids, removal of the embedding extracellular matrix followed by suspension culture elicits rapid morphologic conversion from an apical-in to an apical-out configuration. The resultant relocation of ACE2 to the organoid exterior then allows facile infection by SARS-CoV-2^13^ or entry by bacteria requiring apical access^42^.

**Figure 3.**
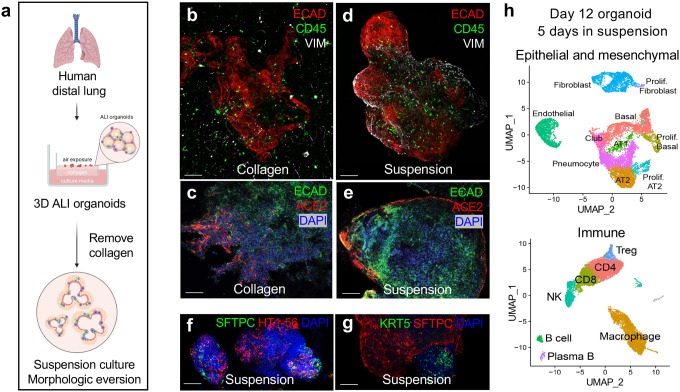
Suspension culture of lung ALI organoids. **a,** Schematic of ALI lung suspension organoid culture. **b,** IF imaging of un-everted day 15 organoids in collagen, ECAD (red), CD45 (green), Vimentin (VIM) (white). Scale bar, 100 μm. **c,** IF imaging of un-everted day 15 organoids in collagen, ECAD (green), ACE2 (red), DAPI (blue). Scale bar, 100 μm. **d-g,** IF imaging of everted day 15 organoids, 5 days in suspension. **d,** ECAD (red), CD45 (green), VIM (white). Scale bar, 100 μm. **e,** ECAD (green), ACE2 (red), DAPI (blue). Scale bar, 100 μm. **f,** AT1 and AT2 cells, HT1–56 (red), SFTPC (green), DAPI (blue). Scale bar, 100 μm. **g,** Basal and AT2 cells, KRT5 (green), SFTPC (red), DAPI (blue). Scale bar, 100 μm. **h,** scRNA-seq UMAP plots showing integrated data of CD45^−^ (top) and CD45^+^ (bottom) cells from suspension organoids, merged from two individual donors, day 12, with 5 days in suspension.

To better simulate the endogenous host-viral interface in which the pathogens such as SARS-CoV2 interface directly with the alveolar epithelium, we performed organoid eversion of 3D ALI lung organoids by ECM removal and suspension culture (Fig. 3a). In collagen, lung ALI organoids contained epithelial cells in central primary structures surrounded by ramifying mesenchyme (Fig. 1c–d and 3b), accompanied by baseline interior expression of ACE2 (Fig. 3c). Upon collagen removal from 7-day established lung ALI organoids and subsequent suspension culture, the epithelium relocated to the surface within 48 hours **(Extended Data Fig. 6a-d)** while preserving mesenchymal and immune cells (Fig. 3d). Notably, suspension culture exposed the ACE2-expressing apical surface of epithelial cells on the external surface of the organoids without obstruction by layers of stroma and collagen matrix (Fig. 3e) while retaining major epithelial cell types such as AT1, AT2, and basal cells in distinct domains (Fig. 3f–g). Single cell RNA-sequencing on CD45^−^ and CD45^+^ populations from suspension organoids confirmed preservation of all major epithelial, stromal and immune cell types (Fig. 3h, **Extended Data Fig. 6e, f).**

### Extended SARS-CoV-2 infection of human lung ALI organoids

**Figure 4.**
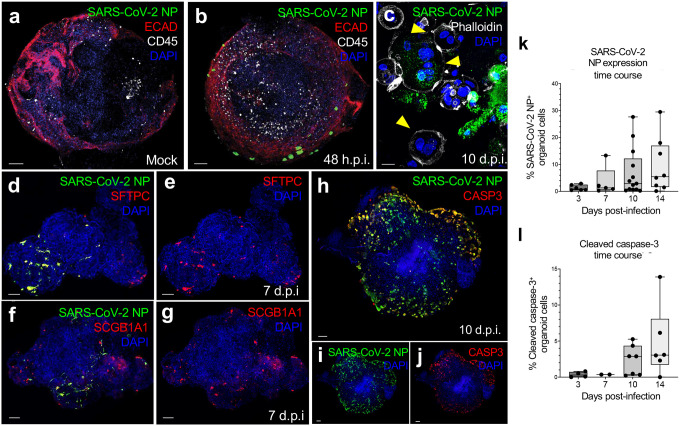
SARS-CoV-2 infection induces apoptosis in suspension lung ALI organoids. **a-b,** IF imaging of mock (uninfected) (a) and SARS-CoV-2-infected (b) suspension lung ALI organoids. SARS-CoV-2 nucleocapsid (NP) protein (green), ECAD (red), CD45 (white), DAPI (blue). 48 hours post-infection with SARS-CoV-2 WA1. Scale bar, 100 μm. **c.** IF imaging of suspension lung ALI organoid with multinucleated syncytia (yellow arrowheads), 10 d.p.i. with SARS-CoV-2, SARS-CoV-2 NP (green), phalloidin (white), DAPI (blue). Scale bar, 10 μm. **d-e.** IF imaging of infected AT2 cells at 7 d.p.i. SFTPC (red), DAPI (blue), with (d) and without (e) SARS-CoV-2 NP channel (green). Scale bar, 100 μm. **f-g.** IF imaging of infected club cells at 7 d.p.i. SCGB1A1 (red), DAPI (blue), with (f) and without (g) SARS-CoV-2 NP channel (green). Scale bars 100 μm. **h-j.** IF imaging of SARS-CoV-2-induced apoptosis, 7 d.p.i., SARS-CoV-2 NP (green), cleaved caspase-3 (red), DAPI (blue). (i) and (j) are different channel splits of (h). Scale bar, 100 μm. **k-l.** Box plot quantification of IF images from **(h-j)**. NP^+^ cells (top) or cleaved caspase-3^+^ cells (bottom) as fraction of total cells in suspension organoids, 3–14 d.p.i. Lines represent lower quartile, median, and upper quartile, whiskers represent data range minima or maxima.

ALI lung organoids were then applied to the longitudinal study of early (2–3 days) and late (14 days) infection with WA-1 SARS-CoV-2. At 48 hours post-infection (h.p.i.), SARS-CoV-2 nucleocapsid (NP) was readily detectable in ECAD^+^ epithelium (Fig. 4a, b). SARS-CoV-2 induces membrane fusion of infected cells and multinucleate syncytia in lung parenchymal cells, visible in autopsy samples from patients who have succumbed to severe COVID-19^43,44^. Accordingly, syncytia formed within infected organoids at later time points (Fig. 4c). Similar to the suspected tropism of SARS-CoV-2^45^, infected cells mostly consisted of AT2 cells (Fig. 4d, e) and, to a lesser extent, club cells (Fig. 4f, g, **Supplementary Table 2)**^13,18,34^. Rare ciliated cell infection was observed in older organoids (>28 days culture) **(Extended Data Fig. 6h)** when these cells were present in relative abundance, in agreement with previous studies^33,34^. SARS-CoV-2-infected NP^+^-cells progressively expressed the apoptotic marker cleaved caspase-3, consistent with end-stage cytotoxicity^33,46^, beginning at days 10 and 14 post-infection (Fig. 4h–j). The proportion of infected cells within a given organoid increased throughout the course of the incubation period. At later time points, over 80% of cells expressing SARS-CoV-2 NP also expressed cleaved caspase-3, indicating frequent progression to cell death (Fig. 4k–l).

### Lung ALI organoids mount innate immune responses to SARS-CoV-2

**Figure 5.**
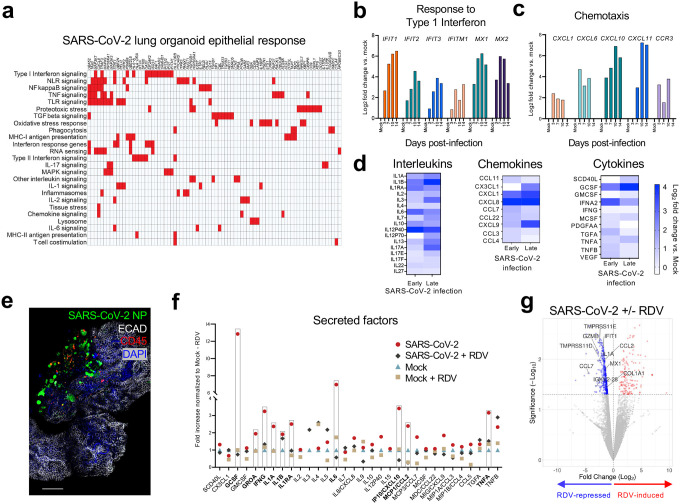
Innate responses to SARS-CoV-2 in lung ALI organoids. **a,** Summary of Nanostring nCounter analysis of upregulated genes and biological pathways (red) in FACS-purified EPCAM^+^ lung epithelial cells from SARS-CoV-2-infected organoids at 7 d.p.i. versus mock control (>1.5-fold change in three biological replicates). **b-c,** Bulk RNA-sequencing of mock and infected organoids over 3–14 days of infection showing genes involved in response to interferon (b), and chemotaxis (c). Y-axis is log_2_ fold change expression normalized to mock of the same time point. **d,** Heat map of Luminex analysis of interleukins (left), chemokines (middle), and cytokines (right), secreted into media by organoids from one representative patient during early (3 days) or late (10 days) of infection. Color corresponds to log_2_ fold change compared to mock of the same time point. **e,** IF imaging of immune cells clustering around infected cells, 10 days post-infection, SARS-CoV-2 NP (green), ECAD (white), CD45 (red), DAPI (blue). Scale bar, 100 μm. **f,** Dot plot of Luminex analysis of secreted factors in culture supernatant after SARS-CoV-2 infection +/− remdesivir (RDV). Red dot: SARS-CoV-2 48 hours post-infection, no RDV. Grey diamond: SARS-CoV-2 48 hours post-infection + 10 μM RDV. Blue triangle: mock infection (no virus), no RDV. Tan square: mock infection (no virus) + 10 μM RDV. Factors increased >2 fold in infected, no RDV over mock, no RDV are bolded and highlighted with rectangles. Y-axis is fold increase normalized to mock (–) RDV condition. RDV was added simultaneously with SARS-CoV-2. **g,** Volcano plot of bulk RNA-seq analysis depicting genes downregulated (blue) or induced (red) upon RDV treatment (10 μM) of SARSCoV-2 infected lung ALI organoids, 48 hours post-infection. Y-axis is -log_10_(p value), x axis is log_2_(fold change normalized to SARS-CoV-2 infection, no RDV).

To analyze innate responses to SARS-CoV-2 infection, we performed quantitative Nanostring digital enumeration of >800 probes recognizing immune-related transcripts in FACS-sorted EPCAM^+^ cells from three biological organoid replicates. Upon confirmed epithelial infection **(Extended Data Fig. 7a)**, 105 out of 820 RNA transcripts were upregulated (>1.5 fold change), associated with multiple immune response pathways including cytokine signaling, inflammasome activation, and antigen presentation (Fig. 5a), alongside strongly increased SARS-CoV-2 genome copy number **(Extended Data Fig. 7b)**. Bulk RNA sequencing of suspension organoids at 3, 7, 10, and 14 days post-infection revealed up to 2^6^-fold time dependent promotion of interferon-stimulated genes such as *IFIT3, MX1* and *MX2*, consistent with an innate antiviral response^47,48^ (Fig. 5b, **Extended Data Fig. 7c)**. Innate chemokines also underwent time-dependent induction, with *CCL10* and *CCL11* remaining particularly elevated (Fig. 5c, **Extended Data Fig. 7c)**.

Additionally, suspension organoid supernatants from early (3 days post-infection) and late (10 days post-infection) timepoints were analyzed by Luminex (Fig. 5d, **Extended Data Fig. 8a-c)**. SARS-CoV-2 promoted secretion of numerous cytokines, with rapid upregulation of IL-6 (2^3^ fold), a key cytokine implicated in COVID-19 severity^5,6^, while the anti-inflammatory cytokine IL-10^49,50^ decreased over time. IL-1α and IL-1ß induction was initially modest during early infection but increased at late stages, potentially exacerbating inflammation^6,51,52^. Diverse chemokines such as CXCL1 and CXCL8, innate immune cytokines such as GCSF (2^4^-fold), IFNA2 (2^5^-fold), and factors implicated in pulmonary fibrosis such as PDGFAA and TGFA^53,54^ were also induced. Accompanying these chemotactic signals, at late infection stages, immune cells could be observed clustering around foci of SARS-CoV-2 in approximately 15% of infected organoids (Fig. 5e, **Extended Data Fig. 8d-e)**.

SARS-CoV-2-induced immune responses were also strongly blocked by the viral replication inhibitor remdesivir^55^, confirming specificity for viral replication. Remdesivir-mediated inhibition was verified by plaque assays using organoid supernatants **(Extended Data Fig. 8f)**. Luminex analysis confirmed that remdesivir potently blocked SARS-CoV-2-induced cytokine release from ALI lung organoids at 48 hours post-infection, particularly evident for GCSF, IFNG, IL6, CXCL10, CCL2, and TNFA (Fig. 5f). Corroborating results were also obtained by organoid qRT-PCR, where remdesivir again strongly inhibited SARS-CoV-2-stimulation of numerous type I and II interferon mRNAs, *TNFA* and *IL6* **(Extended Data Fig. 8g)**. Moreover, bulk RNA-seq of suspension lung ALI organoids revealed substantial remdesivir reversal of SARS-CoV-2-stimulated increases in interferon-stimulated genes (*IFIT1, MX2*) cytokines (*IL1A, IL1B*), chemokines (*CCL7, CCL2*), *GZMB*, associated with cytotoxic T-cell degranulation, and *CD8A*. Loci exhibiting the strongest stimulation by infection and repression by remdesivir included several proteases (*TMPRSS4, TMPRSS6, TMPRSS11D, TMPRSS11E)* closely related to TMPRSS2, a host factor during SARS-CoV-2 receptor-mediated endocytosis^41,56^ (Fig. 5g, **Extended Data 8h-d)**. Rarely, CD68^+^ macrophages contained SARS-CoV-2 NP as detected by immunofluorescence **(Extended Data Fig. 8j)**, resulting from either primary infection or secondary phagocytosis.

### Adaptive immune responses to SARS-CoV-2 in organoids

The adaptive memory of lung-resident lymphocytes both defends against repeated infection and can exacerbate pathologic inflammation as in ARDS and cytokine storm^11^. In marked contrast to epithelial-only conventional organoids^13,17,18^, the lung ALI organoid co-preservation of diverse epithelial lineages and resident immune subsets afforded a unique opportunity to study tissue-resident SARS-CoV-2 adaptive immunity, including T_RM_ cells. Further, the organ-autonomous nature of this system enabled study of lung-intrinsic memory responses in isolation from secondary lymphoid organs.

**Figure 6.**
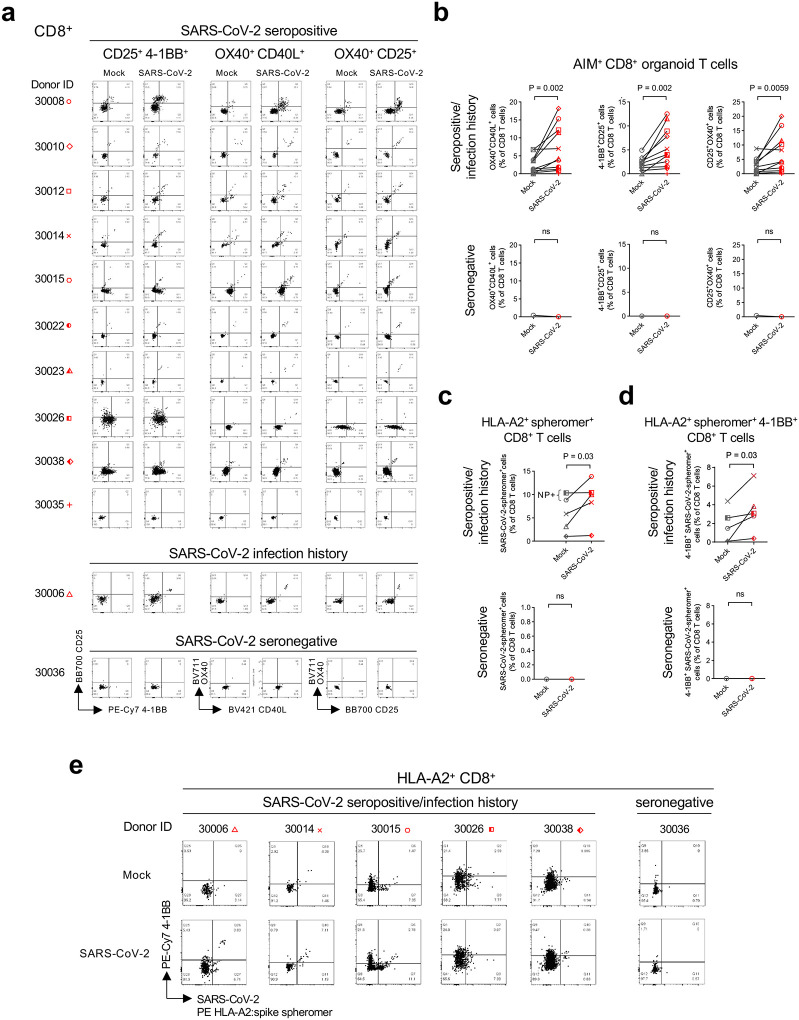
Organoid adaptive T cell responses to SARS-CoV-2 infection. **a-b,** Identification of human ALI lung organoid SARS-CoV-2–responding CD8^+^ T cells by AIM assay. Suspension organoids were infected with SARS-CoV-2 virus for 6 days and responding CD8^+^ T cells were identified based on induction of CD25, OX40, 4–1BB, and CD40L, stratified by SARS-CoV-2 seropositivity and/or infection history of the respective donor individuals. **a,** Individual results for 12 donors, each represented by a corresponding symbol. **b,** Summary results from (a), Wilcoxon matched-pairs test. **c-e,** Identification of SARS-CoV-2– specific CD8^+^ T cells in suspension human ALI lung organoids from HLA-A2^+^ donors using HLA-A*02:01 SARS-CoV-2-spheromers. **c,** Percentage of organoid SARS-CoV-2 spheromer^+^ CD8^+^ T cells out of total CD8^+^ T cells, stratified by SARS-CoV-2 overall seropositivity and/or infection history of the respective donor individuals. Summary results of organoids from 5 seropositive/positive infection history donors and one seronegative donor; SARS-CoV-2 NP-positive serologies are denoted, Wilcoxon matched-pairs test. **d,** Percentage SARS-CoV-2 spheromer^+^ and 4–1BB^+^ double-positive CD8^+^ T cells out of total CD8^+^ T cells from the donors in (c). Summary results of 5 seropositive/positive infection history donors and one seronegative donor. **e,** Flow cytometry plots of 4–1BB^+^ and SARS-CoV-2 spheromer^+^ double positive CD8^+^ T cells from (c) and (d). All gating strategies are shown in **Extended Data Fig. 9a**.

We thus measured SARS-CoV-2-specific adaptive immune responses in T cells from virus- versus mock-infected suspension organoids from seropositive individuals **(Supplementary Table 1)**. We first confirmed SARS-CoV-2 seropositivity in 10 organoid donor individuals (aged 17 months-77 years), all of which exhibited plasma anti-spike (S) upon quantitative electrochemiluminescence assay, consistent with prior vaccination and/or infection. A subset of donor serologies (3/10) exhibited positive nucleocapsid protein (NP) IgG, consistent with prior infection, which could not be excluded in the remaining samples given the established time-dependent decreases of NP titers^57–59^. In the 11^th^ case (30006), plasma was not obtainable at the tissue biopsy, but the medical history confirmed previous SARS-CoV-2 infection. An additional organoid donor (age 7 months) (30036) was seronegative for S, RBD and NP IgG. SARS-CoV2-specific T_RM_ have been previously studied by extraction from tissue, culture with PBMC and peptide megapools, and analysis of T cell receptor–dependent activation-induced markers (AIMs)^3,8^. Agonistic antiCD3 antibody induced 4–1BB, CD25, OX40 and CD40L in organoid CD8^+^ cells, confirming their validity as AIM markers in the culture system (**Extended Data Fig. 9a-b)**. Accordingly, SARS-CoV-2 infection increased the prevalence of 4–1BB^+^CD25^+^, OX40^+^CD40L^+^ and OX40^+^CD25^+^ AIM marker double-positive CD8^+^ T cells in lung ALI organoids from seropositive and/or positive infection history donors, but not from the seronegative donor (Fig. 6a–b, **Supplementary Table 1)**. SARS-CoV-2 also induced OX40^+^CD25^+^ CD4^+^ T cells in a subset of seropositive organoids and not in the seronegative sample, but this did not reach statistical significance **(Extended Data Fig. 9c)**.

To directly detect SARS-CoV-2-specific organoid T cells, we used soluble MHC class I HLA-A2 ectodomain spheromers loaded with SARS-CoV-2 spike and open reading frame (ORF) peptides^60^. These spheromers allowed FACS detection of virus-reactive CD8^+^ T cells within the HLA-A2^+^ organoids used for AIM analysis. The baseline prevalence of spheromer-positive SARS-CoV-2-specific CD8^+^ T cells was higher in organoids from 5 HLA-A2^+^ SARS-CoV-2-seropositive and/or positive infection history donors than from an HLA-A2^+^ seronegative donor (Fig. 6c, **Supplementary Table 1)**. Further, the two HLA-A2^+^ organoid cultures with the highest plasma NP titers (cases 30015 and 30026) exhibited the greatest baseline percentage of SARS-CoV-2-specific CD8^+^ T cells (Fig. 6c, **Supplementary Table 1)**. SARS-CoV-2 infection generally increased the prevalence of spike spheromer-positive CD8^+^ T cells in organoid cultures of HLA-A2^+^ SARS-CoV-2-seropositive and/or positive infection history donors (P=0.03), but not in the seronegative donor. SARS-CoV-2-induced spheromer-positive CD8^+^ T cells were again more prevalent in organoids from seropositive and/or positive infection history than in the seronegative control (Fig. 6c). Additionally, within seropositive and/or positive infection history organoid SARS-CoV-2-spheromer^+^ CD8^+^ T cells, SARS-CoV-2 infection stimulated expression of the AIM markers 4–1BB, CD25, OX40 and CD40L versus mock controls, which did not occur in the seronegative donor organoids (Fig. 6d–e, **Extended Data Fig. 10a)**. The seronegative organoid T cells were still responsive to TCR signaling as indicated by anti-CD3/anti-CD28 induction of 4–1BB and to a lesser extent CD25 **(Extended Data Fig. 10b).** SARS-CoV-2 also induced IFNG expression within organoid SARS-CoV-2-spheromer^+^ CD8^+^ T cells **(Extended Data Fig. 10c)**.

## DISCUSSION

Studies of tissue-resident immune responses are hampered by both inaccessibility relative to peripheral blood and a lack of *in vitro* systems that model infectious agents together with epithelium and resident immune cells as a non-reconstituted entity^2^. Conventional adult lung or iPSC-derived organoids typically contain epithelium but exclude immune compartments^13–19,34,61–64^. Here, we developed an 3-D air-liquid interface human lung organoid system retaining multiple epithelial lineages (basal, club, ciliated, AT1, AT2) as spatially distinct yet adjacent alveolar and airway architectural domains alongside resident mesenchyme (fibroblasts, endothelium). Crucially, human lung ALI organoids preserved endogenous immune cells including CD4^+^ helper, CD8^+^ cytotoxic, *FOXP3*^+^ regulatory T cell subsets, B lymphocytes, plasma cells and macrophages as a cohesive unit, with T cells manifesting memory and tissue-resident phenotypes consistent with T_RM_. Organoid immune content progressively declined but was substantially sustained by the memory T cell-tropic cytokines IL-7 and IL-15; myeloid cells persisted > 40 days without additional cytokine support. The present retention of diverse tissue-resident immune subsets contrasts markedly with prior in vitro strategies, which are both reconstitutive and typically restricted to peripheral blood lymphocytes^32^.

Notably, lung ALI organoids robustly enabled long-term SARS-CoV-2 epithelial infection within a human holistic immune microenvironment. This was greatly facilitated by suspension culture to achieve an apical-out configuration with externally displayed ACE2, analogous to our prior eversion of simpler epithelial-only lung and gastrointestinal organoids^13,42^. The extent of SARS-CoV-2 infection in lung ALI organoids strongly exceeds that of our prior epithelial-only lung organoid system^13^, accompanied by time-dependent apoptosis in infected cells and immune cell chemotaxis to infected foci. Extension to even longer time points could allow modeling of SARS-CoV-2-induced pulmonary failure as relevant to “long-COVID” syndromes^53^.

A salient feature of the current 3D lung ALI method is the recapitulation of innate and, significantly, adaptive immune responses to SARS-CoV-2. The strong viral induction of numerous innate cytokines such as type I interferons was fully anticipated from prior studies of SARS-CoV-2-infected epithelial-only organoid cultures^14,17,18^. However, lung ALI organoids from seropositive and/or positive infection history individuals also manifested adaptive immunity to in vitro SARS-CoV-2 infection in CD8^+^ T cells, with induction of (i) cell surface AIM markers 4–1BB, OX40, CD40L and CD25 and (ii) the presence, expansion and activation of SARS-CoV-2 peptide:MHC spheromer-reactive T cells, consistent with an anamnestic virus-specific memory response. We cannot unequivocally attribute organoid T cell responses to prior infection versus immunization because of time-dependent declines in clinical SARS-CoV-2 NP titers^57–59^. However, since high-level organoid SARS-CoV-2-specific T cell content correlated with NP seropositivity, and since lung T_RM_ have been attributed to local infection^3,65–67^, prior SARS-CoV-2 exposure of donors likely underlies the presence of virus-reactive T_RM_ in organoids, with possible contributions from vaccination^68,69^.

The diversity of tissue-intrinsic immune cells in the current lung organoids provided a unique opportunity to study pulmonary memory T cell responses in the complete absence of secondary lymphoid organs, versus in vivo animal models where these two partitions obligately communicate^4^. Here, the seropositivity-dependence of organoid SARS-CoV-2 adaptive T cell responses suggest that after initial antigen priming, the lung is fully sufficient to compartmentalize and conduct antigen processing, presentation and anamnestic immunity in the absence of a peripheral lymphoid component. This supports postulated roles for local antigen presentation in lung-resident immune memory^39,70,71^ and indeed, lung ALI organoids expressed a complement of genes and cell types relevant to antigen presentation including alveolar macrophages, AT2 cells, endothelium and B cells^39,70^.

Prior analysis of T_RM_ in mice has utilized diverse strategies including parabiosis, transplantation, differential labeling, or inhibition of trafficking, which are not applicable to humans^1,2,4^. In vitro, human T_RM_ have been purified from organs and stimulated with peptide pools, revealing SARS-CoV-2-specific responses^3,66^. The present organoid method offers an adjunctive strategy with physiologic SARS-CoV-2 epithelial infection, antigen presentation and measurement of adaptive responses all within a native lung context. Our studies explore only the acute initiation of adaptive recall and do not exclude possible peripheral lymphoid clonotype replacement during exhaustion following chronic memory T cell responses, by analogy to clonotype exchange in cancer anti-PD-1 immune checkpoint blockade^72^.

Overall, the generation of adult human lung organoids that preserve diverse immune populations *en bloc* with epithelium and mesenchyme represents an enabling method for the *in vitro* study of innate and, significantly, tissue-resident adaptive immunity during pathogen infection. Beyond lung-intrinsic anamnestic responses, this system should be readily adaptable to other organ systems, co-culture with peripheral lymphoid tissues^73^, and extension to emergent infectious agents beyond SARS-CoV-2.

## METHODS

### Human distal lung culture

Fresh human tissues and corresponding blood were obtained as deidentified samples from surgical discards from Stanford Health Care (Stanford, CA). All experiments utilizing human tissue were approved by the Stanford University Institutional Review Board. Standard informed consent for research was obtained in writing prior to tissue procurement and all studies followed relevant guidelines and regulations. Human distal lung was defined as peripheral lung tissue within 1 cm of the visceral pleura. For patients with suspected lung cancer, cases with clinical T4 (American Joint Cancer Committee 6th edition) disease (e.g. features such as bronchial invasion or parenchymal satellite nodule/ metastases) were deferred. Normal tissue was harvested from the lung margin most anatomically distal to palpably well-defined lesions, or from uninvolved lobes in the case of pneumonectomies. Samples with tumors containing ill-defined margins were deferred. Tissue was either processed fresh or placed at 4°C overnight and processed the following morning. The distal lung tissue was washed with PBS, minced finely on ice, and resuspended in Cultrex Rat Collagen I (R&D, 3443–100-01). Next, 1 ml of tissue-collagen suspension was layered on top of pre-solidified 1 ml collagen gel within a 30 mm inner transwell, 0.4 μm pore size (Sigma, PICM03050). After fully solidifying, the collagen transwells were placed in a standard 6-well tissue culture plate (Corning, 353046). 1 ml of lung ALI culture media (see below) was added into the tissue culture plate, below the bottom surface of the collagen-containing transwell. Media was changed twice a week.

### Lung ALI culture media

Advanced DMEM/F12 (Invitrogen, 12634–010) was supplemented with 10 mM nicotinamide (Sigma, N0636), 1 mM n-acetyl cysteine (Sigma, A916S), 1X B-27 supplement minus vitamin A (Gibco, 1258001), recombinant human NOGGIN (100ng/mL, R&D Systems, 120–10C), recombinant human EGF (50ng/mL, R&D Systems, AF-100–15), TGF-beta inhibitor A83–01 (100 nM, Tocris, 2939), penicillin streptomycin glutamine (500 μg/mL, Gibco, 10378–016), normocin (50 mg/mL, InvivoGen, ant-nr-2), HEPES (1 mM, Gibco, 15630–080), and GlutaMAX (1X, Gibco, 35050–061). This mixture was then supplemented with 10% fetal bovine serum (R&D Systems, S11550), 10 μM Y-27632 (Peprotech, 1293823), and 10 μM CHIR 99021 (R&D Systems, 4423).

### Eversion and suspension culture of human lung ALI organoids

Lung ALI organoids were grown as previously described in collagen for 5–10 days. To evert, collagen was removed using collagenase type IV (Worthington, LS004210) for 30 min with shaking at 37°C. Collagenase was washed and quenched with FBS containing media for 3 × 10 min at RT. Organoids were collected by centrifuging at 100 × g for 3 min at RT and resuspended in lung ALI media (above) and plated in 1.5–2mL each in a low-attachment 6-well plate (Corning, 3471).

### SARS-CoV-2 infection of suspension lung ALI organoids

All SARS-CoV-2 work was performed in a class II biosafety cabinet under BSL3 conditions at Stanford University. VeroE6 cells were obtained from ATCC and maintained in supplemented DMEM with 10% FBS. SARS-CoV-2 (USA-WA1/2020) was passaged in VeroE6 cells in DMEM with 2% FBS. Titers were determined by plaque assay on VeroE6 cells using Avicel (Sigma, 11365–1KG) and crystal violet (Fisher, C581–25), viral genome sequence was verified, and all infections were performed with passage 3 virus. Human lung ALI organoids were grown in collagen for 5–10 days, placed into suspension for 2–5 days, counted, then infected with SARS-CoV-2 prior to day 14 in culture. Organoids were resuspended in virus media or an equal volume of mock media, at a MOI of 1 relative to total organoid cells in the sample, and then incubated at 37°C under 5% CO_2_ for 2 h. Organoids were then plated in suspension in lung ALI organoid media. At the indicated timepoints, organoids were washed with PBS for downstream analysis. For remdesivir experiments (RDV), organoids were infected with SARS-CoV-2 as described above and spiked with 10 μM RDV at infection unless otherwise noted.

### Serial brightfield imaging

Tissue culture plates containing the ALI organoids in transwells were imaged serially with a Keyence BZX710 microscope. Images were stitched using BZ-Wide viewer software.

### Histologic analysis of ALI organoids

Collagen from transwell containing ALI organoids were fixed in 10% formalin for 1hr at RT, cut into thin slices, and placed into a histology cassette with 70% ethanol. The collagen was then paraffin embedded and sectioned (4–5 mm). Sections were deparaffinized and stained with H&E for histological analysis.

### Whole-mount staining and confocal microscopy of ALI organoids

Collagen-containing ALI organoids were cut away from the transwell and fixed in 4% PFA for 1 hour at RT. PFA was neutralized with 1X PBS-glycine (130 mM NaCl, 13.2 mM Na2HPO4, 3.5 mM NaH2PO4, 100 mM Glycine, in PBS at pH 7.4) for 30 min at RT, then blocked and permeabilized with 10% donkey serum (Jackson Immunoresearch, 017–000-121) in a permeabilizing solution (130 mM NaCl, 13.2 mM Na2HPO4, 3.5 mM NaH2PO4, 7.7 mM NaN3, 15 μM BSA, 2% Triton X-100, 0.5% TWEEN-20, in PBS at pH 7.4) for 2 hours at RT. Organoids were then stained with primary antibodies at RT for 3 days overnight, followed by 3 X 30 min washes with the permeabilizing solution. Secondary staining used fluorescent donkey secondary antibodies (1:1000, Jackson Immunoresearch) and DAPI for 4 hours at RT, then washed 3 X 30 min with the permeabilizing solution. Organoids were then mounted on slides with mounting buffer (Prolong Gold Antifade mounting media, ThermoFisher Scientific, P36934). Images were acquired using a Zeiss LSM900 confocal microscope and viewed in 3-D using Imaris software. All antibodies, including secondaries, used for immunofluorescence are listed in **Supplementary Table 3**.

### Immunofluorescence analysis of infected organoids

Infected organoids and corresponding mock-infected organoids were centrifuged and washed with PBS. Organoids were then suspended in 4% PFA for fixation and inactivation of virus for 1 hr at RT. PFA was then removed, and organoids were washed with PBS and removed from BSL3 conditions and subjected to whole-mount immunofluorescence staining as described above. All SARS-CoV-2 work was performed in a class II biosafety cabinet under BSL3 conditions at Stanford University. Human lung ALI organoids infected with SARS-CoV-2 were fixed and stained as described above, and images were acquired on a Zeiss LSM900 confocal microscope. The number of infected cells were quantified using the ‘analyze particles’ tool of FIJI (Fiji is just ImageJ) software on MacOS. Briefly, for each sample a five-slice image stack was acquired via the ZEN (blue edition) Microscope software and processed with the Z project tool on FIJI. Image channels were separated and converted to grayscale. Threshold and exposure levels were then set based on images of the mock condition and held constant across all images. The analyze particles tool was utilized to count the number of signals present in each channel, with a size restriction set from 5 (particle units) to infinity, and all other parameters set to default.

#### FACS analysis of immune surface markers from lung ALI organoids:

Collagen-containing lung ALI organoids were digested with Collagenase Type IV (Worthington, LS004210), for 30 min with shaking at 37°C, then centrifuged and washed with lung organoid media containing FBS to quench collagenase. Organoids (now dissociated from collagen) were digested to single cells with Liberase TL (Sigma, 631547) and DNAse (Worthington, LS006328) for 30 min with shaking at 37°C and washed and quenched with FACS buffer (5 mM EDTA + 5% FBS). Cells were stained for viability with Zombie Aqua (Biolegend, 77143) 1:500 in FACS buffer for 20 min on ice, protected from light. After washing with FACS buffer, cells were stained for surface markers with antibodies listed below, all at 1:100. Compensation was performed using OneComp eBeads^™^ Compensation Beads (Thermo, 01–1111-42) and primary antibodies 1:400. Sorting and analysis were performed on a BD FACSAria II SORP, with further data analysis in FlowJo. All antibodies used for FACS are listed in **Supplementary Table 4**.

### Cytokine treatment

Lung ALI organoids were generated as above and lung organoid media was supplemented with the following: recombinant human IL-2 (100 IU/ ml, Peprotech, #200–02), IL-7 (10 ng/ml, Peprotech, #20007), and IL-15 (10 ng/ml, Peprotech, #200–15). After 10, 20, 33, 44, and 65 days of treatment, organoids were dissociated and analyzed by FACS as described above.

### RNA extraction from infected organoids

SARS-CoV-2-infected organoids were inactivated by adding 1000 μl DNA/RNA Shield (Zymo Research, R1100–50) or TRIzol (Thermo Fisher Scientific, 15596018) by incubating for 30 min at RT to decontaminated from the BSL3 facility. RNA was purified using an RNA Clean & Concentrator-25 kit (Zymo Research, R1017) per manufacturer instructions. All RNA samples were treated with DNase (Turbo DNA-free kit, Thermo Fisher Scientific, E1010).

### qPCR analysis of infected organoids

RNA from SARS-CoV-2-infected organoids was extracted by adding 750 μl TRIzol (Thermo Fisher Scientific, 15596018) and purified using an RNA Clean & Concentrator-25 kit (Zymo Research, R1017). All RNA samples were treated with DNase (Turbo DNA-free kit, Thermo Fisher Scientific, E1010). The Brilliant II SYBR Green QRT-PCR 1-Step Master Mix (Thermo Fisher Scientific, 4367659) was used to convert RNA to cDNA and amplify specific RNA regions on the CFX96 Touch real-time PCR detection system (Bio-Rad). RT was performed for 30 min at 50°C, 10 min at 95°C, followed by two-step qPCR with 95°C for 10 seconds and 55°C for 30 seconds, for a total of 40 cycles. All primer sequences are listed in **Supplementary Table 5**.

### Bulk RNA sequencing

RNA library preparations, sequencing reactions, and bioinformatic analysis were conducted at GENEWIZ/Azenta Life Sciences LLC. (South Plainfield, NJ, USA) as follows:

#### Library Preparation

Ultra-low input RNA sequencing library was prepared by using the SMART-Seq HT kit for full-length cDNA synthesis and amplification (Takara, 634436), and Illumina Nextera XT (Illumina,) library was used for sequencing library preparation. Briefly, cDNA was fragmented, and adaptors added using transposase, followed by limited-cycle PCR to enrich and add index to the cDNA fragments. The sequencing library was validated on the Agilent TapeStation (Agilent Technologies), and quantified by using Qubit 2.0 Fluorometer (ThermoFisher Scientific) as well as by quantitative PCR (KAPA Biosystems).

#### Sequencing

The sequencing libraries were multiplexed and clustered onto a flow cell, which after clustering was loaded on an Illumina HiSeq 2500 instrument according to manufacturer’s instructions. The samples were sequenced using a 2×150 Paired End (PE) configuration. Image analysis and base calling were conducted by the HiSeq Control Software (HCS). Raw sequence data (.bcl files) generated from Illumina HiSeq was converted into fastq files and de-multiplexed using Illumina’s bcl2fastq 2.20 software. One mismatch was allowed for index sequence identification.

#### Analysis

After demultiplexing, sequence data was checked for overall quality and yield. Then, sequence reads were trimmed to remove possible adapter sequences and nucleotides with poor quality using Trimmomatic v.0.36. The trimmed reads were mapped to the Homo sapiens GRCh38 reference genome available on ENSEMBL along with the SARS-CoV-2 Wuhan strain reference using the STAR aligner v.2.5.2b. The STAR aligner uses a splice aligner that detects splice junctions and incorporates them to help align the entire read sequences. BAM files were generated as a result of this step. Unique gene hit counts were calculated by using featureCounts from the Subread package v.1.5.2. Only unique reads that fell within exon regions were counted. After extraction of gene hit counts, the gene hit counts table was used for downstream differential expression analysis. Using DESeq2, a comparison of gene expression between the groups of samples was performed. The Wald test was used to generate p-values and log_2_ fold changes. Genes with adjusted p-values < 0.05 and absolute log_2_ fold changes > 1 were called as differentially expressed genes for each comparison.

##### Single-cell RNA sequencing of human lung ALI organoids:

Organoids were harvested either from collagen or suspension, digested into single cells as previously described, and sorted on a BD FACSAria II SORP for singlet discrimination, followed by live/dead and CD45 gating. Cellular suspensions were loaded on a Chromium Single Cell Controller instrument (10x Genomics, PN-000204) to generate single-cell GEMs. Libraries for sequencing was prepped as per manufacturer instructions using a 5’ library prep kit (10x Genomics, PN-1000263). Sequences from scRNA-seq were processed with Cell Ranger (v.3.0.2) software (10x Genomics) with UMI (unique molecular identifier) collapsing and alignment to the GRCh38 human transcriptome. scRNA-seq data from lung tissue and organoids were loaded into Seurat objects following their standard pipeline^74^. Data were filtered with nFeature_RNA values set to >200 and <3500, and percent. Mt values set to <20. For integration, datasets were anchored together with 3,000 integration features and 30 dimensions for identifying anchors before being clustered in accordance with the standard Seurat pipeline. Cell subset gene expressions were analyzed to identify the phenotypic identity of the cell clusters and visualized with Violin and Feature plots in Seurat.

### T cell receptor sequencing analysis

TCR sequences from lung organoids were obtained in parallel with scRNA-seq using the 10x Chromium Single Cell Human TCR Amplification Kit (10x Genomics, PN-1000252) and analyzed with immunarch^75^. Unique TCR clonotypes were defined and quantified based on CDR3b amino acid sequences and V gene segments. We quantified shared TCR clonotypes based on CDR3b amino acid sequences between lung organoid culture and two public TCR databases of known viral antigen specificities, including ImmuneCODE^76^ and VDJDB^77^ databases.

### Luminex from cell culture supernatant

Mock and infected organoids were centrifuged, and supernatant was collected. All supernatants were mixed with 10% Triton-X100 to a final concentration of 1% Triton-X100 for inactivation of virus, decontaminated from the BSL3 facility and frozen at −80°C. Supernatants were analyzed neat and in duplicate using MILLIPLEX^®^ MAP Human Cytokine/Chemokine/Growth Factor Panel A 48 Plex (Millipore Sigma, HCYTA-60K-PX48) by the Human Immune Monitoring Core at Stanford University.

### SARS-CoV-2 spheromer staining

HLA-A*02:01 SARS-CoV-2-spheromers were conjugated with PE and the following spike protein epitopes (ORF1ab3467, ORF1ab4032, ORF1ab4725, S976, S983) were used at 1:10 in FACS buffer^60^. Surface staining antibodies were added at 1:100 and live/dead Fixable Near IR 780 staining (ThermoFisher, L34992) were added at 1:1000. Cells were then fixed with Cytofix (BD, 554714) and permeabilized with 0.5% saponin and stained with the following antibodies at 1:100 dilution: anti-human CD40L, anti-human OX40, anti-human CD137 (antibody details in **Supplementary Table 4**). Data were acquired with BD FACSymphony^™^ A5 Cell Analyzer and analyzed with FlowJo. HLA-A2-expression on organoids used for this analysis was confirmed by FACS.

### Donor SARS-CoV-2 IgG antibody detection

Plasma samples collected from organoid donors were tested with Meso Scale Diagnostics (MSD) electrochemiluminescence (ECL) MULTI-SPOT 96-well plate SARS-CoV-2 assays and instrumentation by following the manufacturer’s recommendations. Briefly, V-PLEX Coronavirus Panel 1 kits (Meso Scale Diagnostics, K15362U-2) were used to detect IgG, IgA or IgM antibodies to SARS-CoV-2 full-length spike, spike receptor-binding domain (RBD), and nucleocapsid antigens. Plasma samples were analyzed at a 1:5000 dilution, detected with SULFO-TAG ECL-labelled anti-human IgG, IgA or IgM, and read with a MESO QuickPlex SQ 120 instrument.

### Nanostring analysis

Organoids were infected with SARS-CoV-2 and dissociated and stained for FACS as described above in a BSL3-certified biosafety cabinet. Cells were fixed with 4% PFA for 1 hr at RT to inactivate virus as described above for decontamination out of the BSL3 facility. Cells were sorted for live, single cell, EPCAM^+^CD45^−^ cells using a BD FACSAria II SORP and RNA was extracted using the protocol described by Russell et. al^78^. Following RNA extraction, quality was assessed via Agilent Bioanalyzer and cDNA was synthesized using Hs HostReponse LI Primers (Nanostring, XT-HHR-12), Coronavirus Panel Plus (Nanostring, CORONAPP-12) and Low RNA Input Kit (Nanostring, LOW-RNA-48) per manufacturer’s instructions. Following hybridization, transcripts were quantitated using the nCounter^®^ SPRINT Profiler. Samples were run by the LCA Genome Core at University of California, San Francisco.

### Statistics and reproducibility

Unless stated otherwise, all data are representative of at least two independent experiments with each independent experiment carried out using an organoid culture derived from a unique individual. Box plot bounds span first through third quartiles, horizontal lines represent median values, and whiskers represent data range minima or maxima. Specific statistical tests used are named in the legend of each figure.

## Data Availability

scRNA-seq datasets have been deposited in Gene Expression Omnibus (https://www.ncbi.nlm.nih.gov/geo/query/acc.cgi?acc=GSE216049) with the accession code GSE216049. This includes raw, processed, and FINAL Seurat objects with all annotations. Bulk RNA-seq datasets (raw and processed files) have been deposited in Gene Expression Omnibus (https://www.ncbi.nlm.nih.gov/geo/query/acc.cgi?acc=GSE230398) with the accession code GSE230398.
